# Next-Generation Biopesticides for the Control of Fungal Plant Pathogens

**DOI:** 10.3390/plants15020312

**Published:** 2026-01-20

**Authors:** Younes Rezaee Danesh, Nurhan Keskin, Solmaz Najafi, Harlene Hatterman-Valenti, Ozkan Kaya

**Affiliations:** 1 Department of Plant Protection, Faculty of Agriculture, Van Yüzüncü Yıl University, 65090 Van, Türkiye; 2 Department of Horticulture, Faculty of Agriculture, Van Yüzüncü Yıl University, 65090 Van, Türkiye; keskin@yyu.edu.tr; 3 Department of Field Crops, Faculty of Agriculture, Van Yüzüncü Yıl University, 65090 Van, Türkiye; solmaznajafi@yyu.edu.tr; 4 Department of Plant Sciences, North Dakota State University, Fargo, ND 58102, USA; h.hatterman.valenti@ndsu.edu; 5 Erzincan Horticultural Research Institute, Republic of Türkiye Ministry of Agriculture and Forestry, 24060 Erzincan, Türkiye

**Keywords:** biopesticides, fungal pathogens, molecular strategies, microbial control, sustainable agriculture, plant pathology

## Abstract

This review explores the innovative approaches in the development of next-generation biopesticides, focusing on molecular and microbial strategies for effective control of fungal plant pathogens. As agricultural practices increasingly seek sustainable solutions to combat plant diseases, biopesticides have emerged as a promising alternative to chemical pesticides, offering reduced environmental impact and enhanced safety for non-target organisms. The review begins by outlining the critical role of fungal pathogens in global agriculture, emphasizing the need for novel control methods that can mitigate their detrimental effects on crop yields. Key molecular strategies discussed include the use of genetic engineering to enhance the efficacy of biopesticides, the application of RNA interference (RNAi) techniques to target specific fungal genes, and the development of bioactive compounds derived from natural sources. Additionally, this review highlights the potential of microbial agents, such as beneficial bacteria and fungi, in establishing biocontrol mechanisms that promote plant health and resilience. Through a comprehensive review of recent studies and advancements in the field, this manuscript illustrates how integrating molecular and microbial strategies can lead to the development of effective biopesticides tailored to combat specific fungal threats. The implications of these strategies for sustainable agriculture are discussed, alongside the challenges and future directions for research and implementation. Ultimately, this review aims to provide a thorough understanding of the transformative potential of next-generation biopesticides in the fight against fungal plant pathogens, contributing to the broader goal of sustainable food production.

## 1. Introduction

Fungal pathogens represent some of the most destructive biotic threats to global agriculture, causing substantial yield reductions that in some cropping systems may reach up to 60% [1]. These losses pose a critical barrier to global food security, an issue that is becoming increasingly urgent as climate change, population growth, and land degradation intensify pressures on agricultural production [1,2,3]. Despite advances in plant protection, current control strategies for fungal diseases face numerous complex constraints. Beyond public concerns regarding chemical inputs, the implementation of strict regulations has reduced the availability of conventional fungicides, many of which previously formed the cornerstone of fungal disease management [2,3,4]. Additional challenges include the rapid emergence of fungicide-resistant pathogen populations, restrictions on certain active ingredients or crop genotypes, and growing evidence of negative impacts on human health and ecosystem stability [4]. In response to these limitations, the development of innovative biopesticides has attracted significant attention as a sustainable alternative in modern agriculture [4,5]. These products employ biocontrol-based mechanisms that either target specific genomic components of fungal pathogens or suppress key pathogenic activities that enable fungi to colonize the plant microbiome. Recent advances have broadened the scope of biopesticidal strategies, leading to more effective agents capable of disrupting early infection stages such as spore germination, hyphal elongation, and tissue colonization [4,6,7]. Despite their promising potential, however, the commercial adoption and field-scale deployment of biopesticides that directly interfere with pathogen development remain relatively limited. This gap highlights the continuing need for rigorous research, farmer-oriented training, and policy support to accelerate the integration of biopesticides into mainstream crop protection programs [8]. Enhancing their adoption could play a vital role in strengthening crop resilience and securing food production under diverse agricultural environments.

## 2. The Challenge of Fungal Plant Pathogens

**Figure 1:**
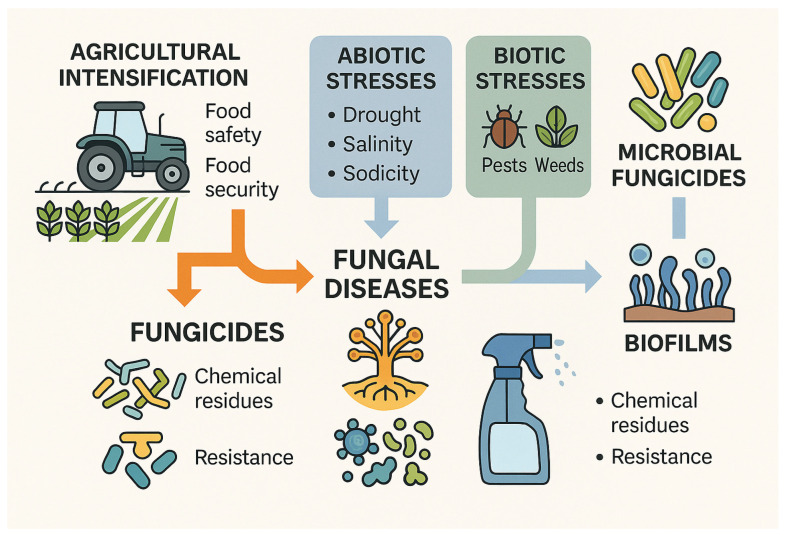
Conceptual overview of global agricultural intensification and its associated challenges. Since the 1970s, increased agricultural production has improved food safety and food security; however, it has simultaneously escalated abiotic stresses (drought, salinity, sodicity) and biotic stresses (fungal, bacterial, and nematode pathogens, pests, and weeds). Fungal pathogens remain the leading cause of global crop losses, driving heavy reliance on chemical fungicides. Overuse of fungicides results in chemical residues, environmental contamination, and fungicide resistance. Increasing awareness of ecological and health concerns is promoting a transition toward microbial fungicides and biologically based disease management strategies. Understanding fungal biofilms offers further opportunities for sustainable plant disease control.

The rapid and substantial expansion of global agriculture since the 1970s has contributed significantly to improvements in food safety, food security, and national food sovereignty. This agricultural intensification has played a central role in feeding a growing global population and improving nutritional quality worldwide [1,2,5]. However, this progress has also been accompanied by an escalation in abiotic stresses—such as drought, salinity, and sodicity—and biotic stresses, including pathogens, pests, and weeds, all of which threaten crop productivity at large scales [1,2,3]. Among biotic threats, fungal pathogens consistently rank as the foremost cause of global crop losses, followed by bacterial pathogens and plant-parasitic nematodes. This pattern was evident during the period of 1990–2012, when assessments of pre-harvest yield losses highlighted the severe economic impacts of biotic stresses [1,9]. Notably, global crop losses caused solely by fungal pathogens were estimated at approximately USD 59 billion, with nearly half of this financial burden incurred in major agronomic crops, underscoring their profound influence on agricultural productivity [1]. To manage fungal diseases, farmers worldwide rely heavily on fungicides. However, extensive and repeated application has resulted in significant chemical residues on crops and accumulation in soils, waterways, and food chains, raising major environmental and public health concerns [3,5]. Growing consumer awareness of food safety issues, coupled with increasing recognition of the ecological damage associated with chemical-intensive agriculture, has intensified the demand for low-toxicity and environmentally benign disease control options. As a result, conventional fungicides are gradually being replaced by microbial fungicides that better align with sustainable agricultural principles [5]. Moreover, many fungal pathogens do not exist as solitary cells but instead form biofilms—structured microbial communities exhibiting coordinated behavior and enhanced resilience. Understanding fungal biofilm biology opens promising avenues for innovative disease control strategies [10,11]. Nevertheless, knowledge of fungal biofilms remains limited, and chemical fungicides remain the predominant approach for plant disease management. The accelerating emergence of fungicide-resistant strains, combined with mounting ecological and health concerns, underscores the urgent need for green, sustainable, and biologically informed disease management strategies that can ensure long-term protection of crops and ecosystems [12] (Figure 1).

## 3. Principles of Next-Generation Biopesticides for Fungal Disease Control

**Figure 2:**
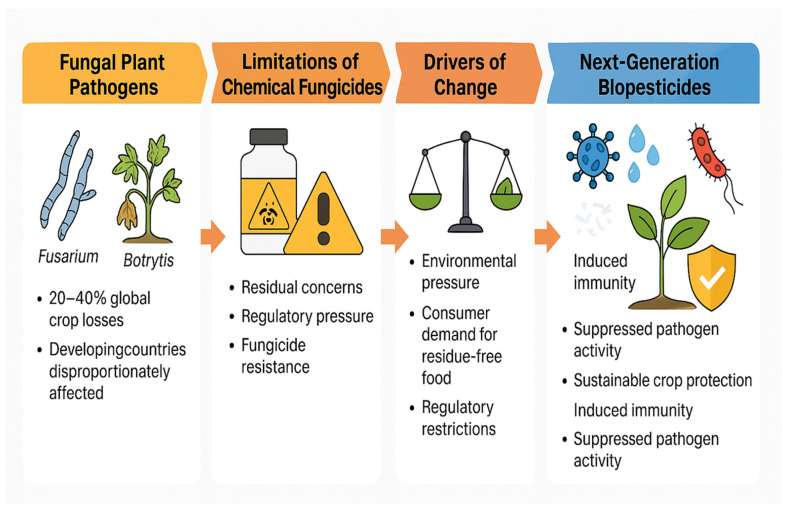
Conceptual representation of the shift from traditional fungicide-based disease management toward next-generation biopesticides. Fungal pathogens such as *Fusarium* and *Botrytis* cause 20–40% global crop losses, disproportionately affecting developing countries. Reliance on chemical fungicides is increasingly constrained due to residue concerns, regulatory pressure, and the rapid emergence of resistant pathogen populations. Environmental demands, regulatory restrictions, and consumer preference for residue-free food drive the transition toward sustainable alternatives. Next-generation biopesticides—based on beneficial microorganisms, molecular elicitors, and antagonistic microbes—offer enhanced plant immunity, suppressed pathogen activity, and long-term sustainable crop protection.

Fungal plant pathogens constitute one of the most critical threats to global crop production, causing average yield and quality losses estimated at approximately 20–40% annually across major cropping systems [2]. Within the diverse fungal kingdom, ascomycetes represent the most significant group of plant pathogens, with genera such as *Fusarium* and *Botrytis* responsible for extensive damage across cereals, fruits, vegetables, and other horticultural crops [9]. These pathogens possess the capacity to infect, colonize, and toxify a wide range of economically important crops, leading to substantial economic losses and undermining food security. As agriculture increasingly transitions toward more sustainable production systems under environmental and regulatory pressures, there is a growing need for alternative crop protection tools capable of specifically targeting these destructive pathogens. Although conventional fungicides remain an important component of disease management, their long-term effectiveness is challenged by regulatory restrictions, consumer demand for residue-free food, and the rapid emergence of fungicide-resistant pathogen populations [3] (Figure 2). The next-generation biopesticides have emerged as promising and environmentally sound solutions for the sustainable management of fungal plant diseases. These innovative tools rely on integrated biological approaches combining molecular elicitors, beneficial microorganisms, and antagonistic microbes to induce plant immunity and suppress pathogen activity [4,13]. Such multifaceted strategies not only interrupt pathogen reinfection cycles but also enhance the crop’s long-term resilience and protection by strengthening host defense responses and stabilizing beneficial plant-associated microbiomes [14,15]. Ultimately, these new generations of biopesticides represent a transformative step toward sustainable crop protection, offering effective means to mitigate fungal disease pressures while simultaneously promoting the health and stability of agricultural ecosystems worldwide [4,16].

## 4. Molecular-Based Control Strategies

Fungal plant pathogens pose a persistent challenge to global agriculture, motivating the development of innovative and more targeted disease control strategies. A key subset of next-generation biopesticide technologies employs molecular mechanisms that exploit plant–pathogen interactions to achieve highly specific suppression of targeted pathogens. Among these approaches, three strategies have gained particular attention: RNA interference (RNAi) and gene silencing, genome editing tools such as CRISPR/Cas systems, and elicitors that activate plant immune responses. RNA interference (RNAi) is an evolutionarily conserved, sequence-specific gene-silencing pathway mediated by small regulatory RNAs that direct the degradation or translational repression of complementary messenger RNAs. Detection of double-stranded RNA (dsRNA) in the cytoplasm initiates its cleavage into short interfering RNA (siRNA), which is subsequently incorporated into an RNA-induced silencing complex to guide the degradation or translational repression of complementary mRNA. Although RNAi plays a central role in antiviral defense in plants, recent studies have shown that exogenous dsRNA can also trigger silencing of homologous genes in filamentous fungi, a phenomenon referred to as horizontal RNA transfer or cross-kingdom RNAi [17,18]. This discovery has been harnessed for the development of pathogen-specific dsRNA molecules applied through leaf sprays—an approach known as spray-induced gene silencing (SIGS)—which has demonstrated systemic activity and effective suppression of fungal and oomycete pathogens across diverse crops [16,19]. Building on the principle of targeted genetic disruption, genome editing technologies such as CRISPR/Cas have expanded the toolkit for fungal pathogen control. CRISPR components can be introduced directly into fungal pathogens to disrupt essential virulence genes or into host plants to modify susceptibility loci and enhance immunity [20]. In fungal systems, CRISPR-mediated modifications include lethal gene knockouts, alteration of pathogenesis-associated genes, and targeted sequence replacements. In plants, decoy-inspired RNA-guided Cas9 constructs have shown promise for engineering resistance against viruses and potentially other non-target pathogens [21]. These advancements highlight the capacity of genome editing to reshape host–pathogen dynamics at the molecular level. A complementary molecular strategy involves the use of elicitors—bioactive molecules that activate plant immunity by triggering specific signaling pathways. Elicitors can induce systemic acquired resistance (SAR) or prime plants to respond more rapidly and robustly upon subsequent pathogen attack, thereby reducing disease severity [13]. Such priming mechanisms generate a heightened physiological state that can limit pathogen colonization even when the initial infection is non-lethal. Collectively, RNA-based silencing approaches, genome editing systems, and elicitor-mediated priming represent powerful next-generation strategies that may provide durable and pathogen-specific disease management solutions in sustainable agriculture.

### 4.1. RNA Interference and Gene Silencing

RNA interference (RNAi) is an evolutionarily conserved, sequence-specific gene-silencing pathway mediated by small regulatory RNAs that direct the degradation or translational repression of complementary messenger RNAs. In plants, RNAi plays a central role in gene regulation and defense against viruses, transposable elements, and other biotic stressors, including fungal pathogens. In fungal systems, core RNAi components such as Dicer-like, Argonaute, and RNA-dependent RNA polymerase proteins are conserved, enabling cross-kingdom RNA-based interactions relevant to disease control strategies [22,23]. Understanding the molecular architecture of RNAi in both host plants and fungal pathogens is therefore essential for the development of efficient, environmentally friendly RNA-based biopesticides. In plants, RNAi is a well-characterized mechanism in which an antisense RNA signal triggers the generation of double-stranded RNA (dsRNA) precursors, typically 21–25 nt in length, which are processed into 21–24 nt small interfering RNAs (siRNAs) that guide sequence-specific degradation of complementary mRNAs. The dsRNA triggers can originate from intron-containing genes, inverted transgene repeats, or branched RNA (bRNA) generated through RdRP activity on viral RNA, transposable elements (TEs), or genomic inverted repeats [24]. RNA silencing can be activated by diverse biotic or abiotic stimuli, and during viral infections is often manipulated through viral suppressors of silencing, which enhance plant susceptibility. Gene silencing efficiency can also be increased through inverted-repeat transgenes that elevate dsRNA production. However, the development of RNA-based bioprotectants against fungal pathogens has progressed more slowly. Early RNAi systems were conceptualized for antiviral protection and required adaptation before they could be applied to eukaryotic pathogens whose biology is more complex and diverse. Successful deployment of RNA-based biopesticides depends on understanding pathogen uptake mechanisms, dsRNA stability, and delivery routes—whether via host-induced gene silencing (HIGS) in transgenic plants or spray-induced gene silencing (SIGS) using exogenous dsRNA [25]. Recent evidence shows that fungi can naturally internalize environmental dsRNA, which can disrupt cellular metabolism, alter virulence, or silence essential genes, thereby strengthening the rationale for developing RNA-based crop protection strategies as biodegradable, precise, and sustainable alternatives to chemical fungicides. Despite the considerable promise of spray-induced gene silencing (SIGS) as a non-transgenic disease control strategy, its underlying mechanisms and field-level robustness remain under active debate. While early studies demonstrated that exogenous dsRNA can be taken up by fungal pathogens and induce sequence-specific gene silencing, leading to disease suppression [17,18], more recent work has questioned whether observed protective effects are always mediated by canonical RNA interference pathways. In particular, it was reported that disease reduction following dsRNA application may occur independently of sequence-specific RNAi, potentially involving alternative stress responses or non-specific immune activation [26]. These findings highlight the need for caution when interpreting SIGS efficacy solely through an RNAi-centric framework. Consequently, future research should aim to disentangle RNAi-dependent and RNAi-independent mechanisms, improve dsRNA delivery and stability, and validate sequence specificity under field conditions to establish the true potential of SIGS-based biopesticides.

### 4.2. Genome Editing Technologies

Genome editing technologies have transformed modern biology by enabling precise and targeted modifications of nucleobases, thereby expanding the diversity of genomic alterations that can be engineered [27,28,29]. Among these platforms, CRISPR/Cas9 has emerged as the leading technology, revolutionizing how genomic modifications are introduced into plants, fungi, and other organisms. Originally discovered as an adaptive immune mechanism in prokaryotes, CRISPR/Cas systems employ guide RNAs (gRNAs) to recognize and cleave specific DNA sequences, enabling highly customizable editing based on user-designed gRNAs [27,30]. Beyond the CRISPR/Cas9 system, a broad range of CRISPR-based genome-editing tools has been developed, including Cas12a (Cpf1), Cas13 for RNA editing, CRISPR-associated base editors, transcription activator-like effector nucleases (TALENs), and zinc-finger nucleases (ZFNs), each offering distinct capabilities and organism-specific applications, including for fungal pathogens [28]. Genome editing can be directed toward disrupting pathogenicity-associated genes, modifying virulence factors, or altering host susceptibility pathways to confer enhanced disease resistance.

Although off-target effects still remain a significant challenge, multiple improvements have been introduced to increase precision. These include the development of high-fidelity Cas9 variants, advanced computational tools for gRNA design, multiplexed sgRNA delivery systems, and the emergence of prime editing technologies that enable precise changes without double-strand breaks [29]. The implications of genome editing for plant disease control are extensive. CRISPR-based approaches are facilitating fundamental research on fungal pathogenicity, enabling functional characterization of virulence genes, and supporting the development of innovative biological control strategies. Moreover, simultaneous editing of both pathogen effector genes and host resistance genes offers new potential for durable, broad-spectrum disease management. The successful establishment of CRISPR/Cas9-enabled genome editing in fungal pathogens—together with rapid advances in editing crop genomes—opens promising new avenues for sustainable, molecularly informed disease management in agriculture [20,31]. Although CRISPR/Cas-based technologies are widely recognized as powerful tools for functional genomics and mechanistic studies of fungal pathogenicity, their relevance extends beyond fundamental research toward direct disease control applications. Targeted knockout or modification of fungal virulence genes using CRISPR/Cas systems has been shown to significantly attenuate pathogenicity and impair infection processes in experimental systems, thereby providing valuable mechanistic insights into fungal virulence and host–pathogen interactions. While these studies primarily support indirect disease management through target identification and functional validation rather than direct field application, they nevertheless establish an important foundation for the rational design of future control-oriented strategies [20,31]. In addition, genome editing of host susceptibility genes represents a complementary approach to disease management by enhancing resistance without reliance on chemical fungicides [21,29]. In this context, CRISPR-based technologies support molecular disease control by enabling precise disruption of pathogenic mechanisms and informing the rational design of next-generation biopesticides and resistant crop varieties.

### 4.3. Pathogen-Triggered Immunity and Signal Modulation

A detailed understanding of the molecular mechanisms underlying plant–fungal interactions—encompassing both pathogen virulence strategies and plant immune responses—is therefore essential for developing advanced and sustainable molecular approaches for fungal disease control [9,13,32]. Upon invading plant tissues, fungal pathogens deploy a wide array of virulence factors, including cell wall-degrading enzymes (CWDEs), effector proteins, and phytotoxins. These components act synergistically to breach plant barriers, suppress immunity, and facilitate colonization [33]. In response, plants have evolved highly sophisticated immune systems capable of perceiving danger signals in their environment and activating tailored defense responses. At the cellular level, the perception of immune signals triggers rapid production of reactive oxygen species (ROS), shifts in ion fluxes, the accumulation of antimicrobial phytoalexins, and transcriptional activation of immune-related genes [13,32,34]. These responses often culminate in localized hypersensitive cell death, serving to restrict pathogen spread [32,35]. Furthermore, local immune activation can propagate throughout the plant to establish systemic acquired resistance (SAR), which confers long-lasting protection against subsequent infections [14]. Elicitors are key molecular cues—derived from pathogens or plants—that enhance resistance to both biotic and abiotic stresses by activating innate immune responses. Pathogen-associated molecular patterns (PAMPs), such as chitin fragments from fungal cell walls, are recognized by specific pattern-recognition receptors (PRRs) on plant cells, thereby triggering PAMP-triggered immunity (PTI) and downstream defense signaling cascades [32,36,37]. In contrast, the recognition of pathogen effector proteins by intracellular immune receptors activates effector-triggered immunity (ETI), a more robust and often stronger defense response [32]. Recent research has identified numerous fungal elicitors and their corresponding plant receptors, providing critical insight into the mechanisms of elicitor-triggered immunity. This expanding knowledge base lays the foundation for engineering novel fungal elicitors capable of activating durable immune responses in plants, offering exciting possibilities for sustainable agricultural practices [38]. In parallel with these established molecular strategies, recent advances in plant immunity engineering have introduced additional next-generation approaches for fungal disease management. One rapidly developing area is nucleotide-binding leucine-rich repeat (NLR) receptor engineering, which enables the modification, optimization, or stacking of immune receptors to enhance recognition of fungal effector proteins and improve the durability of effector-triggered immunity (ETI) [32,38]. In addition, immune-related and antimicrobial peptides have gained attention as bio-inspired tools capable of directly inhibiting fungal growth or priming host defense responses with high specificity and minimal environmental impact [13,14]. Recent developments in genome editing technologies further extend these concepts by enabling precise modification of host susceptibility genes or pathogen virulence factors, thereby complementing RNA interference- and elicitor-based strategies for targeted disease control [20,21,29]. Collectively, these contemporary approaches reinforce the concept that next-generation biopesticides will increasingly integrate immune engineering, molecular targeting, and microbiome-informed strategies to achieve durable and sustainable fungal disease management. The main characteristics, strengths, and limitations of RNAi, CRISPR/Cas editing, and elicitor-induced immunity are summarized in Table 1.

## 5. Microbial Biopesticides and Microbiome Engineering

Emerging biocontrol developments are increasingly highlighting the role of beneficial fungi and bacteria as effective biological agents for regulating a broad range of fungal pathogens [4,6,7]. Numerous microbial species demonstrate strong antagonistic activity against major crop-infecting fungi such as *Fusarium* spp. and *Botrytis cinerea*, while maintaining compatibility with key agricultural crops including rice, wheat, tomato, tobacco, and grapevine [4,6,7,39,40]. Important performance criteria for evaluating microbial biopesticides include reductions in infection incidence and symptom severity, inhibition of conidial germination on susceptible plant surfaces, suppression of mycelial growth, and decreased accumulation of pathogen-associated mycotoxins in harvested tissues [6]. A particularly promising direction in biocontrol involves root-associated beneficial microorganisms identified and selected using metagenomic and microbiome profiling approaches [6,7,41]. Such microbes enhance plant tolerance to biotic stress, promote growth, and support healthy microbiome assembly. Beneficial rhizosphere fungi and bacteria can significantly mitigate damage inflicted by diverse pathogens and pests, improving plant resilience and productivity [4,6,7]. Advances in metagenomic sequencing have deepened our understanding of the complex interaction networks between plant roots and their associated microorganisms, accelerating efforts toward designing next-generation biopesticides that target critical microbe–plant–pathogen interactions [15]. Furthermore, biocontrol agents that target fungi, insects, or nematodes achieve higher commercial success when selected based on rhizosphere metagenomic traits and ecological fitness. This approach aligns with current regulatory trends that favor biocontrol products derived from native or locally adapted microbial populations. As the field advances, integrating these microbial strategies offers substantial promise for sustainable agriculture by reducing chemical inputs while providing robust and ecologically aligned crop-protection solutions [7].

### 5.1. Beneficial Fungi and Bacteria

One promising alternative to chemical control is the use of beneficial microorganisms—both fungi and bacteria—as biological control agents. These living microbes can suppress pathogen growth, compete for ecological niches, induce plant immunity, or degrade pathogen-derived toxins [4,6,13]. Importantly, microbial biocontrol approaches are environmentally sustainable and compatible with ecological and low-input farming systems [5,7,41]. Numerous commercial products based on antagonistic fungi and bacteria are now widely available, offering practical tools to help farmers reduce reliance on synthetic fungicides [7]. However, the successful implementation of biocontrol strategies requires a comprehensive understanding of the biology, ecology, and interactions between biocontrol agents, plant hosts, and their target pathogens [4,6,39]. Such knowledge ensures that the selected agents are used under optimal conditions and in cropping systems where their activity can be maximized. Several major beneficial fungal and bacterial species—along with their antagonistic mechanisms and associated crop systems—are discussed in subsequent sections. Table 2 summarizes key beneficial microbial taxa, their antagonistic mechanisms, target pathogens, and evidence levels supporting their use as biocontrol agents.

### 5.2. Endophytes and Rhizosphere Dynamics

**Figure 3:**
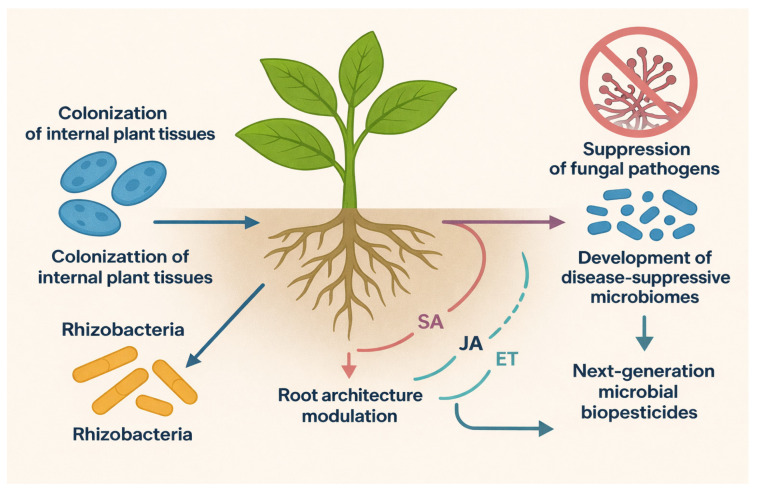
Mechanistic model illustrating the role of endophytes and rhizobacteria in enhancing plant defense and contributing to next-generation microbial biopesticides. Endophytes colonize internal plant tissues and influence root exudation patterns, thereby modulating microbiome assembly and promoting plant resilience. Rhizobacteria regulate root architecture and activate plant immune pathways, including salicylic acid (SA), jasmonic acid (JA), and ethylene (ET). These interactions collectively induce systemic resistance and facilitate the development of disease-suppressive microbiomes, ultimately supporting the suppression of fungal pathogens and the effectiveness of microbial biopesticides in sustainable agriculture.

The development of next-generation biopesticides increasingly focuses on beneficial endophytes and rhizobacteria capable of suppressing a wide variety of fungal pathogens, making them a central topic in sustainable plant protection research [4,6,7]. Endophytic microbes exhibit specialized biological traits that enable them to colonize internal plant tissues following successful establishment within the root zone, where they contribute to disease suppression, immune modulation, and improved plant performance [6,43]. During interactions with plant roots, these microbes influence root exudation patterns, which not only complement the plant’s carbon supply but also play a pivotal role in shaping the composition and diversity of the plant-associated microbiome, thereby enhancing overall plant health and resilience [34,35]. Bioengineering strategies that target endophyte manipulation and rhizosphere microbiome optimization aim to improve plant tolerance and protection against major fungal pathogens. Rhizobacteria, in particular, regulate root architecture through the modulation of auxin dynamics and influence plant immune signaling by interacting with salicylic acid (SA), jasmonic acid (JA), and ethylene (ET) pathways (Figure 3). These mechanisms collectively strengthen plant defenses and contribute to induced systemic resistance [13,36]. Moreover, both endophytes and rhizobacteria aid in identifying and enriching disease-suppressive microbial communities by revealing microbiome assembly principles and enabling the prediction of candidate microbial suppressors associated with specific fungal pathogens. Such advances significantly enhance the potential and reliability of microbial biopesticides in sustainable agriculture.

### 5.3. Metagenomic-Guided Strain Selection

Through the application of metagenomics, researchers can significantly accelerate the screening and selection processes of microbial biopesticides. This advanced approach reveals intricate microorganism–pathogen interaction networks by providing detailed characterization of the active microbial fraction, along with insights into microbial genomes and the various metabolic pathways involved. Consequently, the vast number of plant–microbe interactions that surround cultivated crops and their potential applications for biocontrol can be more accurately predicted and evaluated, opening new avenues for effective agricultural practices [37].

## 6. Formulation, Delivery, and Environmental Fate

**Figure 4:**
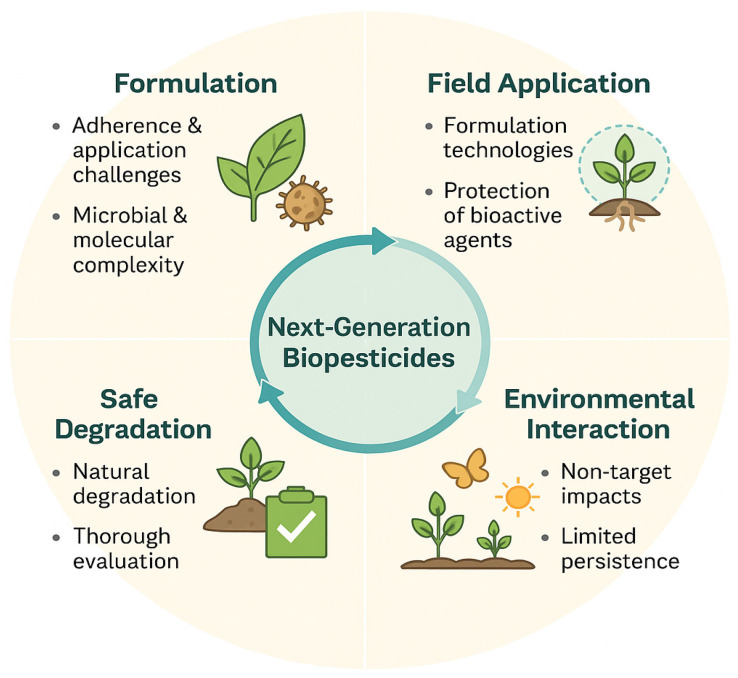
Conceptual cycle illustrating key considerations in the development, application, and environmental behavior of next-generation biopesticides. Successful formulation requires overcoming challenges related to adherence, application, and the inherent biological and molecular complexity of microbial products. Field application depends on advanced formulation technologies that protect bioactive agents under agricultural conditions. Once applied, biopesticides interact with the environment, where non-target impacts and persistence must be carefully evaluated. Ideally, biopesticides undergo safe degradation following their biological function, requiring thorough assessment of environmental fate, biodegradability, and ecological safety.

Next-generation biopesticides are increasingly recognized as a pivotal component of future plant protection strategies, motivating extensive research into their broad potential benefits. A major challenge lies in the development of formulations that adhere effectively to crop surfaces and can be reliably applied using standard agricultural equipment. At the same time, such products must demonstrate environmentally benign behavior, ensuring that they do not pose risks to non-target organisms or disrupt ecosystem function [39]. Microbial and molecular biopesticides exhibit substantial biological and chemical diversity, making their formulation and field deployment inherently complex. A critical factor in their successful implementation is understanding how biopesticides behave when transitioning from controlled laboratory conditions to real agricultural environments (Figure 4). Modern formulation strategies increasingly rely on microencapsulation and polymer-based carriers designed to protect microbial propagules from abiotic stresses such as UV radiation, desiccation, and temperature fluctuations, thereby extending shelf life and preserving bioactivity until application [42]. Stable multimolecular microparticle structures are particularly advantageous because they can safeguard spores or biomolecules during storage and release them effectively once deployed in the field. Despite their promise, the development of such innovative formulations requires considerable time and refinement to ensure long-term stability and reliable performance. Equally important is a thorough understanding of the environmental fate of biopesticides before and after application, as both unwanted persistence and unintended spread may lead to adverse ecological consequences. Potential outcomes include disruption of beneficial microbial communities, selection for resistant pathogen strains, or accumulation in sensitive habitats. Ideally, next-generation biopesticides should exhibit limited persistence in the environment, degrading naturally after achieving their intended biological function. However, assessing persistence, biodegradability, and non-target impacts requires rigorous evaluation and extensive validation under field conditions. Although this process demands substantial effort, it is essential to ensure that biopesticides deliver their intended benefits while maintaining environmental safety and supporting sustainable agricultural systems [44]. In addition to biological and molecular formulation strategies, enabling technologies such as nanotechnology and computational screening tools are increasingly shaping the development of next-generation crop protection agents. Nanomaterial-based delivery systems have demonstrated strong potential to enhance the stability, uptake, and targeted delivery of small RNAs, bioactive molecules, and metal-based agents, thereby improving efficacy while reducing overall application rates and environmental exposure [45,46]. Such nano-enabled platforms are particularly relevant for RNAi-based biopesticides, where protection from degradation and efficient cellular uptake remain key challenges [47,48]. In parallel, structure-based virtual screening and molecular docking approaches are emerging as powerful tools for identifying novel antifungal targets and guiding rational pesticide design, complementing experimental screening pipelines [49]. Together, these enabling technologies support the transition toward more precise, efficient, and environmentally responsible plant protection strategies.

### 6.1. Encapsulation and Formulation Technologies

To enhance the stability of biological agents and prolong shelf life, encapsulation technologies are increasingly applied in formulating both molecular- and microbial-based biopesticides. Conventional pesticides typically degrade quickly upon exposure to various environmental elements, leading to finite effectiveness and an unavoidable risk of loss of quality due to photochemical or hydrolysis processes. Encapsulating the active substance within a polymer, lipid, or silica matrix not only decreases the intrinsic reactivity of the active ingredient but also easily modulates the release profiles [45]. A wide range of synthetic and natural polymeric carriers have been utilized to effectively stabilize several biological molecules. Furthermore, conventional biopesticides may still require various additions of protection agents, carrier materials, and minuscule quantities of classical agrochemicals or assistance agents in the preparation of the suspension solution. These requirements render the preparation and application of biopesticides cumbersome, especially when the active components could have been applied as simple single or double-storage capsules. The *Cunninghamella* species, a prevalent fungus in biocontrol preparations, shows the capability of producing broad-spectrum antifungal and antibacterial agents [39]. To evaluate the preservation of its antifungal activity at different cultivation periods, the obtained culture supernatants at intervals of 1, 10, and 20 days were successfully microencapsulated in an edible gelatin capsule. The shelf-life stability of the encapsulated preparation at 4 °C and room temperature also indicated that anti-lagenidium, anti-sclerotinia, and anti-verticillium activity were still detectable after storage for 30 days. Establishing a series of active component-protected formulations that increase the shelf life of both the biological particles and the extracted suspension not only guarantees fewer time-consuming mills of fungus-crop contact but also effectively enables growing large quantities of the fungus, resulting in a considerably accelerated scaling-up process for biopesticide production.

### 6.2. Application Methods and Field Deployment

To ensure successful delivery through a wide array of agricultural equipment and across diverse production systems, next-generation biopesticides must be formulated with careful attention to commercial standards, regional agronomic practices, and local environmental constraints. Traditional commercial biopesticide formulations often contain 10–20 individual components, including carriers, surfactants, stabilizers, UV protectants, and spreader–sticker additives. However, practical advances in formulation science indicate that 5–15 well-chosen additives are generally sufficient to achieve high stability, effective delivery, and strong biological performance [42]. The optimal timing for biopesticide application depends on the biology of the target fungal pathogen, the crop growth stage, and local cropping calendars. Modern formulations are designed to integrate seamlessly with existing agricultural operations, ensuring that they can be applied alongside conventional farming activities without causing disruption to neighboring management practices. This compatibility is essential for maintaining farm-level ecological balance and for promoting grower adoption, particularly in regions where pesticide application schedules are tightly regulated or seasonally constrained [44].

### 6.3. Persistence, Non-Target Effects, and Risk Assessment

The persistence of biopesticides and their potential non-target effects must be thoroughly assessed using comprehensive and standardized methodologies. Evaluating environmental fate requires examining multiple degradation pathways, including transformation into non-active components, immobilization within soil matrices, and potential dispersal into aquatic systems, to ensure environmental safety and regulatory compliance [5,42,46]. Climate variables such as temperature, humidity, and UV exposure strongly influence the breakdown kinetics of microbial and molecular biopesticides [40]. In many cases, key formulation components remain chemically stable across sequential laboratory and field trials while maintaining high efficacy against their intended target organisms. Importantly, well-designed microbial biopesticides often exhibit high specificity, suppressing target pathogens without causing significant harm to non-target microbial or faunal communities [41]. To ensure effective delivery and biological activity, a minimum persistence period—typically several days—is required to allow for colonization, metabolic activation, and transmission within plant tissues or the rhizosphere. Comprehensive risk assessments must therefore consider exposure pathways, including bioavailability in water, potential ecotoxicity, and transfer into adjacent habitats. Monitoring plans for microbial formulations may require periodic assessments based on environmental conditions, particularly during periods when runoff or drift is likely. As production is scaled to accommodate deployment across larger cropping systems, additional scrutiny of environmental persistence and non-target impacts becomes essential. This includes evaluating formulation stability, dispersal behavior, and ecosystem-level interactions. A multifaceted approach integrating environmental monitoring, ecotoxicological evaluation and adaptive risk management is therefore critical to support the safe and sustainable use of next-generation biopesticides while ensuring protection of non-target organisms and surrounding ecosystems [44].

## 7. Regulatory, Ethical, and Societal Considerations

In Europe, the regulation of biopesticides is governed under the same overarching framework that applies to chemical pesticides, primarily the EU Regulation (EC) No. 1107/2009. As a result, biopesticides are subject to stringent safety and efficacy requirements comparable to those imposed on synthetic chemical active substances. This regulatory structure necessitates comprehensive characterization of both the chemical and biological properties of biopesticidal products. The concepts of environmental safety, toxicological evaluation, and impacts on non-target organisms are applied with equal rigor to microbial and RNA-based biopesticides as they are to conventional pesticides [43]. Although biopesticides offer significant environmental and agronomic advantages, their adoption still poses societal challenges, including public concerns about genetically modified organisms (GMOs) and biotechnology-derived plant protection tools. Current research efforts in both academic and industrial sectors are increasingly focused on the regulatory and legislative challenges associated with RNA interference (RNAi)–based fungicides [47,50]. Strengthening stakeholder engagement—including regulators, researchers, growers, and consumers—is essential for articulating both the benefits and risks of RNAi-based biopesticides. Such dialogue will help shape transparent risk-governance strategies and facilitate broader acceptance during this critical transition toward sustainable, biologically based plant protection systems.

## 8. Final Considerations and Future Perspectives

Direct support for innovative next-generation biopesticide concepts remains limited, with most of the available evidence coming from individual case studies rather than systematic meta-analyses. Trials employing RNA interference (RNAi) sprays or CRISPR/Cas-based strategies often show strong pathogen-specific effects under controlled conditions, attracting considerable scientific interest [46,47]. However, their broader applicability and field robustness have not yet been thoroughly evaluated, and many of the potential risks are inferred rather than empirically excluded through rigorous environmental and multi-site testing [48]. Similarly, while numerous studies report effective colonization and protective activity from fungal endophytes and beneficial rhizobacteria, only a limited number provide robust, quantifiable field-scale data for externally applied microbial agents. In the few cases where such data exist, the observed reductions in disease are typically modest or strongly dependent on environmental conditions such as soil type, moisture, or temperature [51]. Despite major advances in metagenomics, microbial strains that consistently prevent disease under real field conditions have not yet been identified or selected through genome-informed screening pipelines [52]. These gaps may partly explain the currently low interest among biopesticide developers and end-users toward unconventional biological products. A comprehensive meta-analysis integrating available trials would provide substantially stronger evidence for practical use and could stimulate greater commercial investment in these emerging technologies. In the meantime, the persistent lack of field-validated microbial biopesticides underscores the need for scientists to also explore fully synthetic yet biologically inspired plant-protection agents that target alternative molecular pathways, thereby opening new opportunities for innovation in sustainable agriculture.

## 9. Gaps in Knowledge and Future Directions

Major advances have been made in the understanding and control of fungal plant pathogens; however, many obstacles remain before the promise of next-generation biopesticides can be fully realized. The following lists some of the pressing questions that remain open, together with possible approaches to their investigation and the areas in which additional research, technological development, or methodological innovations are urgently needed. The ability of fungal pathogens to communicate, share nutrients, and even exchange DNA suggests that some form of filamentous network is likely to be common across taxa. Exploring the extent of network connectivity that enables such alliances could be informative with respect to how the spread of agricultural threats can be limited [10,11]. Three-dimensional physical models of plants could be combined with hydroponic growth techniques to simulate effects on tissue water and gas exchange that are difficult to quantify in traditional 2D assays. Functional–structural plant models that explicitly represent 3D architecture and hydraulics already provide powerful tools for scaling leaf-level processes to whole-plant and canopy behavior [53,54]. Equally, models that simulate exchanges of elements or nutrients across soil zones, or links between above- and below-ground components, could greatly advance understanding of intrinsic pathogen controls. While advances in transcriptomics, metabolomics and other omics platforms generate large data sets intended to complement or substitute for experimentation in simplified systems, substantial uncertainty remains regarding how best to integrate and interpret such data in complex plant–pathogen pathosystems [55,56]. Classic network-based approaches, including graph theory, cumulative-time-dependent scaling, affinity propagation, correlation clustering, and factor analysis, can help identify common features among diverse tissues or organisms and highlight pathways or substances of particular interest [49]. Confidence in hypotheses can be increased by focusing on sequences, biomolecules, and pathways already supported by independent lines of evidence. Investing in awards, prizes, or challenges that reward young researchers or students at schools or universities as well as mainstream scientists might assist in identifying promising solutions to these and other critical questions.

## 10. Conclusions

Biopesticide strategies discussed in this review are unlikely to function as standalone solutions but instead are best integrated into comprehensive disease management frameworks. When combined with existing agronomic practices, these approaches have the potential to enhance the sustainability and resilience of modern crop protection systems. Successful implementation will require coordinated efforts among researchers, industry stakeholders, funding agencies, and policymakers to accelerate development, regulatory approval, and field adoption. Biopesticide models rooted in naturally occurring ecological mechanisms offer additional advantages by reducing environmental risks and minimizing concerns associated with the introduction of exotic microorganisms. Such approaches may also streamline regulatory pathways and support broader acceptance. Overall, continued interdisciplinary research and collaboration will be essential to translate next-generation biopesticides into practical, effective tools that contribute to sustainable agriculture and long-term food security.

## Figures and Tables

**Table 1:** Comparison of major molecular biopesticide strategies.

Strategy	Mode of Action	Target Specificity	Advantages	Limitations	Key References
RNA interference (SIGS/HIGS)	dsRNA processed into siRNA, silencing essential fungal mRNAs	Very high (sequence-dependent)	Non-GMO option (SIGS), biodegradable, minimal residues	Variable dsRNA uptake, environmental degradation	[16,18]
CRISPR/Cas genome editing	gRNA-directed Cas nuclease disrupts virulence genes or modifies host susceptibility	Very high	Precise edits, durable resistance, powerful research tool	Off-target risks, regulatory challenges	[20,31]
Elicitors (PTI/ETI/SAR activation)	PRR perception triggers immune cascades, systemic resistance	Medium–high	Broad-spectrum protection, non-toxic, already commercialized	Overactivation may reduce growth; variable field performance	[14,38]

**Table 2:** Major microbial biopesticide groups and their mechanisms against fungal pathogens.

Microbial Group	Mechanisms of Action	Target Pathogens	Crop Systems	Evidence Level	References
*Trichoderma* spp.	Mycoparasitism, enzymes, volatile metabolites, ISR induction	*Fusarium*, *Botrytis*, soilborne fungi	Horticultural crops, cereals	Extensive laboratory + moderate field	[4,39]
*Bacillus* spp.	Lipopeptide antibiotics, enzyme secretion, ISR	Broad fungal pathogens	Rice, wheat, maize, tomato	Strong laboratory + increasing field	[7,42]
*Pseudomonas* spp.	Siderophores, antibiotics, biofilm competition	Root pathogens, wilt fungi	Most major crops	Strong laboratory evidence	[6,7]
Endophytic fungi	Colonization of tissues, immune modulation, metabolite production	Opportunistic plant pathogens	Fruit trees and cereals	Growing evidence; variable field	[40,43]
Rhizosphere bacteria	SA/JA/ET modulation, niche competition, growth promotion	Wide range of fungi and oomycetes	Broad cropping systems	Extensive laboratory + moderate field	[6,41]

## Data Availability

No new data were created or analyzed in this study.
